# NF90 coordinately represses the senescence-associated secretory phenotype

**DOI:** 10.18632/aging.100497

**Published:** 2012-10-31

**Authors:** Kumiko Tominaga-Yamanaka, Kotb Abdelmohsen, Jennifer L. Martindale, Xiaoling Yang, Dennis D. Taub, Myriam Gorospe

**Affiliations:** ^1^ Laboratory of Genetics, National Institute on Aging-Intramural Research Program, National Institutes of Health, Baltimore, MD 21224, USA; ^2^ Laboratory of Molecular Biology and Immunology, National Institute on Aging-Intramural Research Program, National Institutes of Health, Baltimore, MD 21224, USA

**Keywords:** RNA-binding protein, ribonucleoprotein complex, mRNA translation, mRNA stability, post-transcriptional gene regulation, senescence

## Abstract

A hallmark trait of cellular senescence is the acquisition of a senescence-associated secretory phenotype (SASP). SASP factors include cytokines and their receptors (IL-6, IL-8, osteoprotegerin, GM-CSF), chemokines and their ligands (MCP-1, HCC4), and oncogenes (Gro1 and Gro2), many of them encoded by mRNAs whose stability and translation are tightly regulated. Using two models of human fibroblast senescence (WI-38 and IDH4 cells), we report the identification of RNA-binding protein NF90 as a post-transcriptional repressor of several SASP factors. In ‘young’, proliferating fibroblasts, NF90 was highly abundant, associated with numerous SASP mRNAs, and inhibited their expression. By contrast, senescent cells expressed low levels of NF90, thus allowing SASP factor expression to increase. NF90 elicited these effects mainly by repressing the translation of target SASP mRNAs, since silencing NF90 did not increase the steady-state levels of SASP mRNAs but elevated key SASP factors including MCP-1, GROa, IL-6, and IL-8. Our findings indicate that NF90 contributes to maintaining low levels of SASP factors in non-senescent cells, while NF90 reduction in senescent cells allows SASP factor expression to rise.

## INTRODUCTION

Most untransformed cells divide in culture for a finite number of times until they reach a state of long-term growth arrest termed senescence [1]. Cellular senescence can be triggered by many factors, including oxidative damage, excessive mitogenic signals, and genotoxic stress caused by telomere erosion [2]. By preventing the propagation of damaged cells that could become tumorigenic, senescence is believed to represent a mechanism of tumor suppression. Senescent cells can remain viable and metabolically active for along time, displaying distinct protein expression patterns that enable a potent growth repressive state. In addition, senescent cells display a characteristic pattern of secreted factors, including proteins with promalignant effects such as the growth-related oncogene (GRO), and factors that promote inflammation, angiogenesis, and degradation of the basement membrane, such as interleukins (IL-6 and IL-8), vascular endothelial growth factor (VEGF), and matrix metalloproteases (MMPs) [3]. This trait of senescent cells has been termed senescence-associated secretory phenotype (SASP) or senescence messaging secretome (SMS) [4,5].

With advancing age, senescent cells accumulate in tissues and the SASP-elicited proinflammatory state is believed to have a complex influence on age-related conditions [6]. For example, two major SASP factors, IL-6 and IL-8, together with other SASP factors, attract immune cells to the tissue in which senescent cells reside; depending on the tissue context, this immune surveillance can promote processes such as wound healing, the resolution of fibrosis, and tumor regression [7-9]. At the same time, SASP factors can compromise the integrity of the ECM, thus facilitating cancer cell migration. In addition, the systemic proinflammatory phenotype seen in the elderly is believed to affect a broad range of age-related pathologies, including diabetes, cancer, neurodegeneration and cardiovascular disease [10] and contributes to an age-related decline of the adaptive immune system (immunosenescence [11]).

Despite the great potential impact of the SASP on the biology of senescence and aging, the mechanisms that regulate SASP are poorly understood. At the transcriptional level, nuclear factor (NF)-κB has been identified as a key regulator of the transcription of SASP factors, including IL-1, IL-6, and IL-8, in cooperation with C/EBP (CCAAT/enhancer binding protein) ß, another transcription factor similarly implicated in the response to stress, immune, and inflammatory signals [12-15]. NF-κB signaling was responsible for establishing SASP in a senescence model of IMR-90 (human skin) fibroblasts expressing H-RasV12 [16]. The NF-κB component RelA/p65 was more significantly enriched in the chromatin of senescent fibroblasts than in that of young, proliferating fibroblasts, and was phosphorylated on Ser536, a positive transactivating modification. Accordingly, lowering p65 prevented the induction of senescence by HRasV12 [16].

The mammalian p38 mitogen-activated protein kinase (MAPK) plays a critical role in the inflammatory responses by promoting the expression of SASP factors such as MCP-1 and MIP-1α [17]. In human fibroblasts, constitutive p38 activity induced SASP by activating the transcriptional function of NF-κB [18]. As reviewed by Salminen and coworkers [19], p38 can activate NF-κB through a number of mechanisms [17], such as by promoting the activity of kinases MSK1 and MSK2, which phosphorylate the NF-κB transactivating subunit p65 and potentiate NF-κB signaling [20-22], by inducing p65 acetylation, another modification that increases the transactivation function of NF-κB [23], and by stimulating histone H3 phosphorylation, which facilitates the recruitment of NF-κB to the promoters of cytokine and chemokine genes like *IL6*, *IL8*, and *CCL2* (encoding IL-6, IL-8, and MCP-1, respectively [24]). These observations link p38 and NF-κB as potent inducers of the expression of SASP factors. Besides p38, the RIG-1 type of the inflammasome receptor was activated in senescent cells and promoted NF-κB activity; these effects were blocked by Klotho, a protein inhibitor of RIG-1. Other activators of SASP factors via NF-κB include the TGF-ß-TAK1 pathway, the HMGB1 protein of the high-mobility group protein family, and ceramides [19].

At the post-translational level, the microRNA miR-146a/b was recently shown to inhibit expression of the IRAK1 (IL-1 receptor-associated kinase 1), a critical component of the IL-1 signal transduction pathway. By reducing IRAK1 levels, miR-146a/b blocked the secretion of IL-6 and IL-8, two main pro-inflammatory SASP factors. Bhaumik and colleagues proposed that in this manner miR-146a/b prevented excessive SASP [25].

Besides regulation of the transcription and secretion of SASP factors, there is evidence that the mRNAs that encode SASP factors are regulated at the post-transcriptional level. Changes in mRNA stability and translation are long recognized to be major regulators of the expression of cytokines, chemokines, growth factors, extracellular matrix (ECM) remodeling enzymes, and other SASP factors [26-29]. Rapid changes in the stability and/or translation status of these mRNAs permit a tight control of the expression of the encoded proteins in response to stress, immune, and inflammatory signals. The post-transcriptional regulators responsible belong to two main classes of molecules: RNA-binding proteins (RBPs) and noncoding RNA (among which microRNAs are the best known group). RBPs implicated in controlling mRNA turnover and translation include human antigen R (HuR), AU-binding factor (AUF)1, KH-type splicing regulatory protein (KSRP), tristetraprolin (TTP), T-cell intracellular antigen-1 (TIA-1 and the related protein TIAR), BRF1, and nuclear factor 90 (NF90) (reviewed in Ref.[30]).

While extensive evidence supports the notion that acute stimuli can affect the expression of individual SASP components, it is not known whether the expression of SASP factors in senescent cells is regulated via post-transcriptional mechanisms. Here, we report the identification of NF90 as an RNA-binding protein that binds to numerous mRNAs encoding SASP factors (collectively named SASP mRNAs) and coordinately influences their post-transcriptional fate in a senescence-dependent manner. In young, early-passage, proliferating fibroblasts, high NF90 levels contributed to the repression of SASP factor production. This repression was elicited mainly via reduction in SASP factor translation through their 3'UTRs. By contrast, in senescent cells NF90 levels were markedly reduced, which allowed increased expression of numerous SASP factors. Our results are consistent with a role for NF90 as a coordinator of the inhibition of SASP factor production in early-passage, proliferating fibroblasts; in senescent cells, the lower levels of NF90 lead to SASP de-repression, permitting higher expression of SASP factors such as MCP-1, GROa, IL-6, and IL-8.

## RESULTS

### Changes in levels of SASP mRNAs with replicative senescence

**Figure 1:**
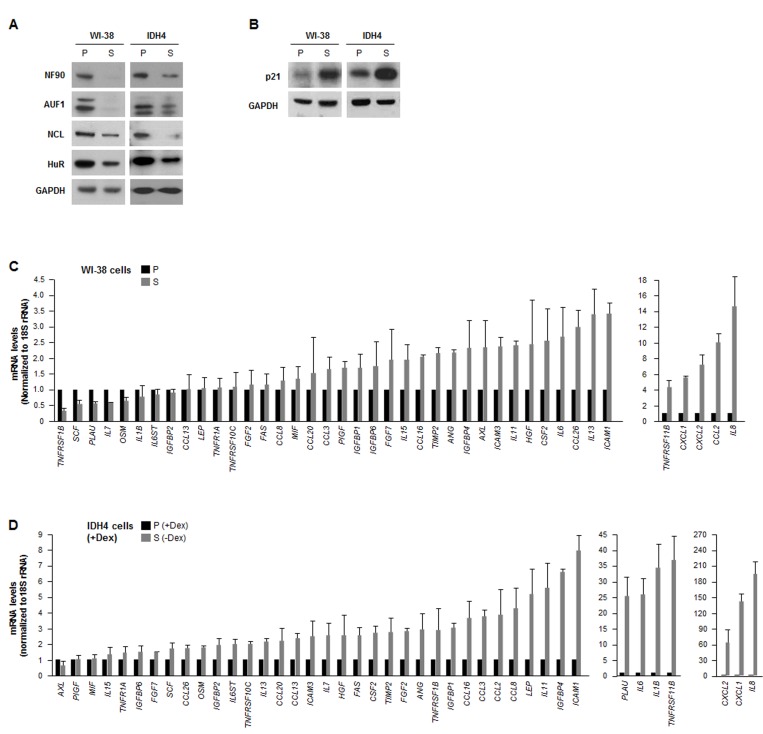
The levels of RBP NF90 are lower in senescent cells **(A)** Western blot analysis of the expression levels of RBPs HuR, AUF1, NCL and NF90 in whole-cell lysates prepared from human diploid WI-38 fibroblasts ['young', proliferating (P, pdl 20) or senescent (S, pdl 54)] and from human IDH4 fibroblasts [P (+Dex) or S (-Dex for 9 days)]; the levels of GAPDH were assessed as a loading control. **(B)** Western blot analysis of the senescence-associated cdk inhibitor p21 in cells from the populations described in panel A. **(C,D)** In the WI-38 **(C)** and IDH4 **(D)** cell populations described in panel A, total RNA was prepared and the levels of mRNAs encoding the SASP factors shown were measured by RT-qPCR. Data are the means and standard deviation (+SD) from three independent experiments.

We investigated the post-transcriptional regulation of the expression of SASP factors in two human fibroblast models: WI-38 and IDH4. We compared ‘young’, proliferating (P), early-passage (population doubling, pdl ~20) WI-38 fibroblasts with late-passage, senescent (S, pdl ~54) WI-38 fibroblasts. We also compared IDH4 fibroblasts, which resembling ‘young’ cells and proliferate rapidly (P) when cultured with dexamethasone (Dex) for stable expression of SV40 large-T antigen, and become senescent (S) by ~9 days after Dex is removed from the culture media. As shown in Fig. 1A, a number of RBPs showed lower expression levels in senescent cells (S, -Dex) than in proliferating cells (P, +Dex). The diminished levels of HuR, AUF1, and nucleolin in senescent cells was previously reported [30-32]. However, the lower levels of NF90 in senescent fibroblasts was a novel finding. The senescence marker p21 was upregulated in senescent WI-38 and IDH4 populations (Fig. 1B).

In parallel, we surveyed a subset of mRNAs expressed in WI-38 and IDH4 cells (each proliferating and senescent, P and S). Reverse transcription (RT) followed by real-time-quantitative (q)PCR amplification was performed using a battery of primers that detected mRNAs encoding SASP products (as described by Coppé et al [4]); the levels of 18S rRNA were also measured and used for normalization. Quantification of the signals revealed that a handful of SASP mRNAs were lower in senescent cells of both cell types, as compared with proliferating cells, but generally SASP mRNAs were either unchanged or higher in senescent cells (Fig. 1C,D), supporting the view that besides higher secretion, SASP factor biosynthesis was also upregulated in senescent cells [4]. The relative fold changes were not identical in both sets of P and S cell types, but both cell groups shared the most highly upregulated transcripts, including *IL8*, *CCL2*, *CXCL1*, *CXCL2*, *TNFRSF11B*,*IL6*, and *ICAM1* mRNAs, encoding IL-8, MCP-1, GROa, GRO(a,b,g), osteoprotegerin, IL-6, and ICAM1, respectively.

### NF90 associates with numerous SASP mRNAs

**Figure 2:**
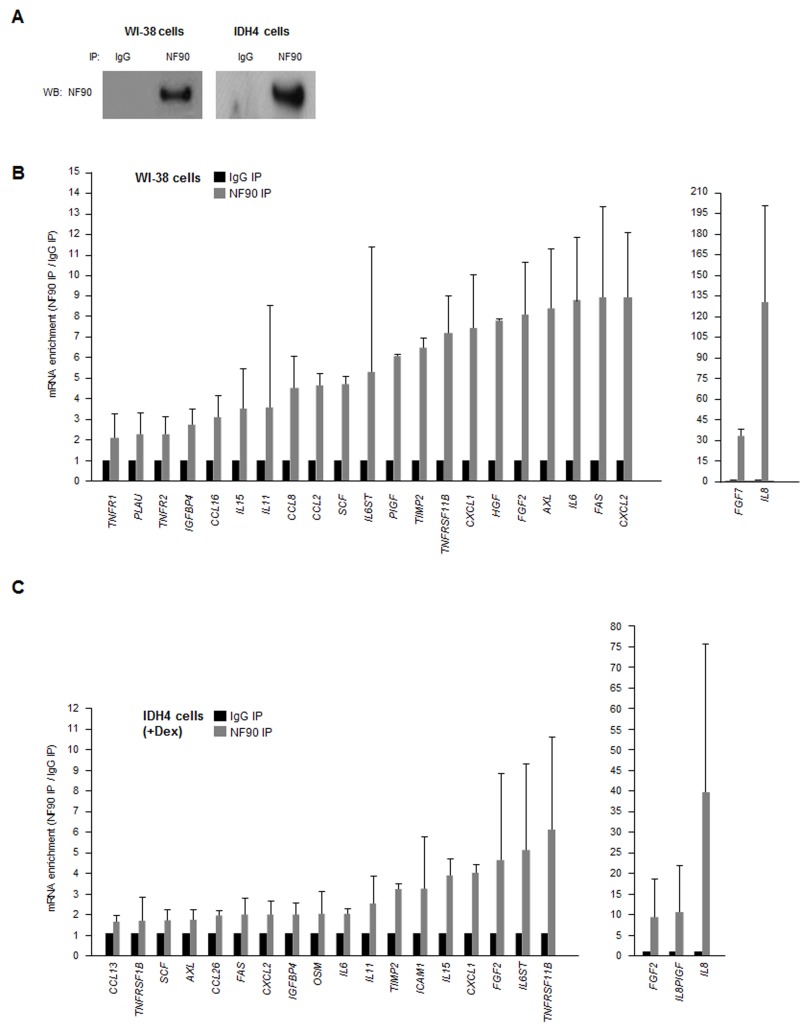
NF90-associated SASP mRNAs **(A)** Lysates were prepared from proliferating WI-38 (P) and IDH4 (+Dex) cells and subjected to immunoprecipitation (IP) using either anti-NF90 or IgG antibodies, whereupon Western blot (WB) analysis was used to detect NF90 levels in the IP samples. **(B,C)** The levels of SASP mRNAs in the ribonucleoprotein (RNP) complexes present in the IgG IP and NF90 IP samples from panel A were measured by RT-qPCR analysis. The levels of enrichment of SASP mRNAs in NF90 IP relative to IgG IP were calculated. Enrichments >2-fold are shown for WI-38 cells **(B)** and enrichments >1.5-fold are shown for IDH4 cells **(C)**. In **(B,C)** data are the means +SD from three independent experiments.

As most SASP mRNAs (Fig. 1C,D) are known targets of various RBPs, we hypothesized that a given RBP might coordinately regulate the fate of several SASP mRNAs. Due to a number of technical and biological limitations we did not pursue analysis of HuR, AUF1, nucleolin or TTP (not shown); instead, we focused on NF90. By ribonucleoprotein (RNP) immunoprecipitation (RIP) analysis using an anti-NF90 antibody under conditions that preserved the integrity of RNPs, NF90 was effectively enriched after IP of both WI-38 and IDH4 lysates (Fig. 2A). The RNA present in the IP was isolated and subjected to RT-qPCR [reverse transcription (RT) followed by real-time, quantitative (q)PCR] analysis to monitor the levels of SASP mRNAs in NF90 IP relative to the control (IgG) IP. The same catalog of transcripts as in Fig. 1C was assayed, but only those mRNAs showing enrichments ≥twofold in the NF90 IP from WI-38 or IDH4 cells are shown (Fig. 2B,C). The most highly enriched NF90 target in both cell lines was *IL8* mRNA, which showed a striking level of enrichment of >100-fold in WI-38 cells (pdl 22) and ~40-fold in IDH4 (+Dex) cells. Other transcripts such as *CXCL1*, *IL6*, *FAS*, and *TNFRSF11B* mRNAs were also enriched in NF90 IP materials obtained from WI-38 and IDH4 cells. These findings indicate that NF90 collectively associates with many mRNAs encoding SASP factors in human fibroblasts.

**Figure 3:**
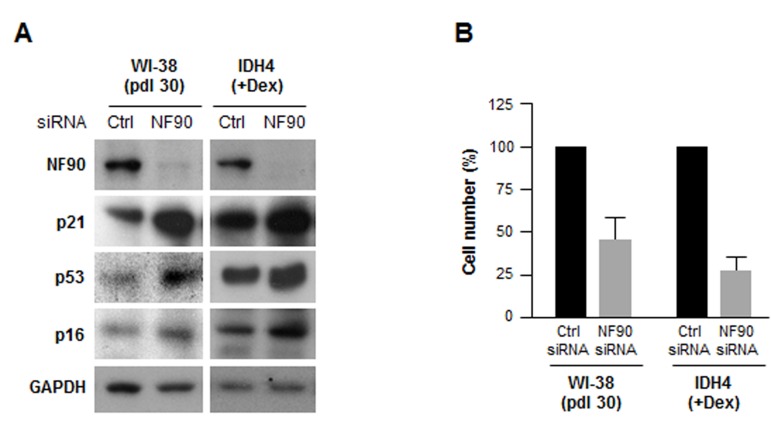
Silencing NF90 increases fibroblast sense-cence, decreases proliferation **(A)** Forty-eight h after transfecting WI-38 cells (pdl 30) and IDH4 cells (+Dex) with either NF90 siRNA or Ctrl siRNA, whole-cell lysates were prepared and the levels of senescence markers p21, p53, and p16 were studied by Western blot analysis. **(B)** WI-38 and IDH4 cells were transfected as explained in panel A; two weeks later cells were counted and plotted. Data represent the means +SD from three independent experiments.

To investigate if NF90 was relevant to fibroblast senescence, we silenced NF90 by using small interfering (si)RNA. As shown in Fig. 3A, 48 h after transfection with NF90 siRNA, NF90 levels were markedly reduced, while the levels of senescence markers p21, p16, p53 were higher, suggesting that NF90 might contribute to repressing or delaying cellular senescence; nonetheless, by 48 h after siRNA transfection, all of the cells were negative for the activity of the senescence marker ß-galactosidase (not shown). Silencing NF90 was growth inhibitory for both WI-38 and IDH4 fibroblasts, as determined by measuring changes in cell number two weeks after siRNA transfection (Fig. 3B).

### NF90 represses the translation of several SASP mRNAs

**Figure 4:**
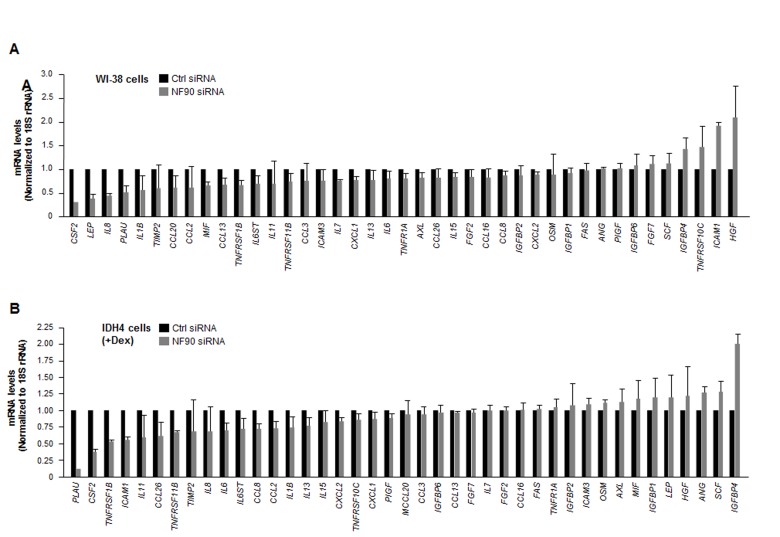
Influence of NF90 on the steady-state expression of SASP mRNAs **(A)** Forty-eight h after transfecting WI-38 cells (pdl 30) with Ctrl siRNA or NF90 siRNA, total RNA was prepared and the levels of SASP mRNAs were quantified by RT-qPCR analysis (normalized to 18S rRNA levels). **(B)** Forty-eight h after transfecting IDH4 cells (+Dex) with Ctrl siRNA or NF90 siRNA, total RNA was prepared and the levels of SASP mRNAs were quantified by RT-qPCR analysis (normalized to 18S rRNA levels). Data represent the means +SD from three independent experiments.

Silencing NF90 in WI-38 cells lowered the steady-state abundance of several SASP mRNAs. Reductions to one-half of their concentration in the NF90 siRNA group relative to the Ctrl siRNA group were seen for *CSF2*, *LEP*, and *IL8* mRNAs in WI-38 cells (Fig. 4A), and for *PLAU*, *CSF2*, and *TNFRSF1B* mRNAs in IDH4 cells (Fig. 4B). The only transcript reduced in both groups was CSF2 mRNA (encoding GM-CSF), an mRNA that did not appear to interact with NF90 (Fig. 2B,C). Regarding transcripts upregulated after NF90 was silenced, only *HGF* mRNA (in WI-38 cells) and *IGFBP4* mRNA (in IDH4 cells), two NF90 targets, showed twofold enrichment. These observations suggested that NF90 did not globally influence the levels of target SASP mRNAs.

**Figure 5:**
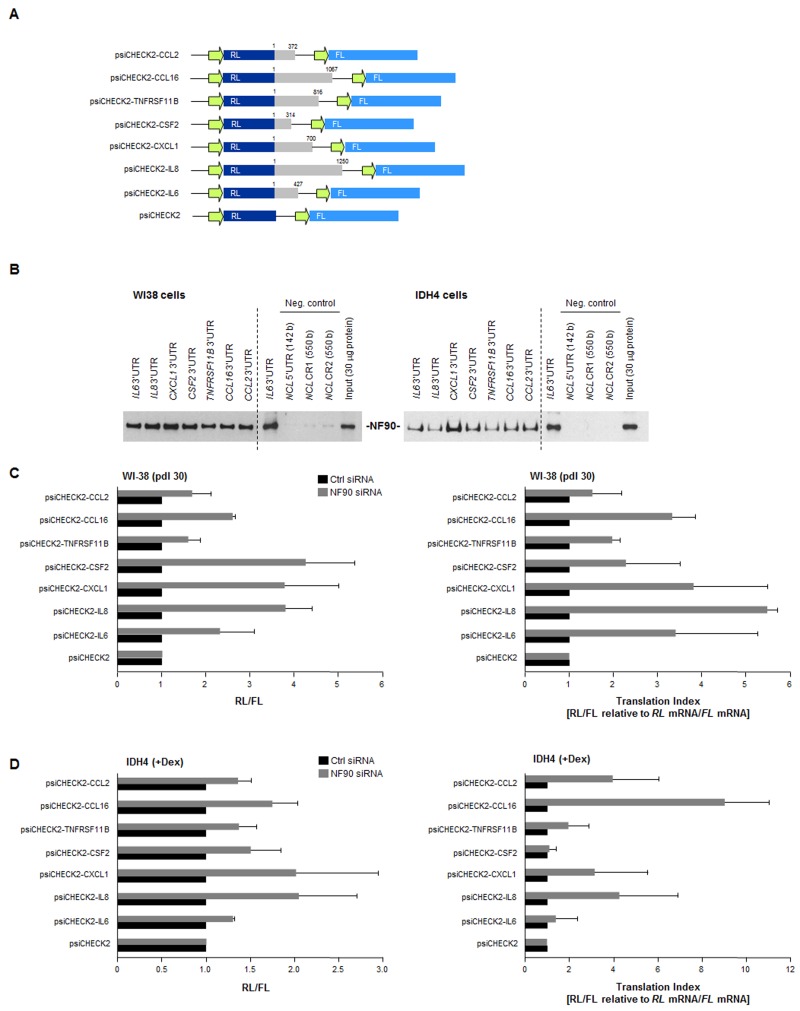
Effect of NF90 on the expression of reporter constructs bearing SASP 3'UTRs **(A)** Schematic of reporters used. Reporter constructs were derived from the psiCHECK2 parent plasmid by insertion of 3'UTRs from *CCL2*, *CCL16*, *TNFRSF11B*, *CSF2*, *CXCL1*, *IL8*, and *IL6* mRNAs (gray) in the 3' end of the renilla luciferase (RL) coding region (dark blue); the internal transfection control firefly luciferase (FL, light blue) was expressed from the same plasmid backbone. **(B)** Biotin pulldown analysis of the interaction of NF90 with the biotinylated 3'UTRs shown in panel A (gray) was carried out as explained in the Methods section. Negative control RNAs spanned the 5'UTR and CR of the nucleolin (*NCL*) mRNA. **(C)***Left*, WI-38 cells (pdl 30) were transfected with Ctrl or NF90 siRNAs; 24 h later, cells were further transfected with each of the plasmids shown [WI-38 cells (800 ng/ml plasmid) and IDH4 cells (8 ng/ml plasmid)] and 16 h after that, the levels of Renilla luciferase were calculated and normalized to the levels of Firefly luciferase in the same transfection group. *Right*, the “translation indeces” were calculated for the reporter constructs. Relative differences in RL/FL were compared with differences in *RL* mRNA/*FL* mRNA, in order to calculate how much of the changes in reporter activity were due to changes in transcript levels (relative to changes in protein levels). **(D)** IDH4 cells were transfected and investigated using the same strategies as described in panel C. In **(C,D)** data are the means +SD from three independent experiments.

To investigate if NF90 affected the translation of target SASP mRNAs, as was shown for a different subset of mRNAs expressed in cervical carcinoma cells [33], we prepared reporter constructs bearing the 3'UTR of target mRNAs, the mRNA region where NF90 preferentially interacts [33]. Constructs were prepared to test the full-length 3'UTRs of *CCL2*, *CCL16*, *TNFRSF11B*, *CSF2*,*CXCL1*, *IL8*, and *IL6* mRNAs using the reporter plasmid psiCHECK2 as backbone (Fig. 5A). Confirmation that NF90 was capable of interacting with these 3'UTR segments was obtained by preparing biotinylated RNA spanning the 3'UTRs of these mRNAs (Methods). After incubation of the biotinylated transcripts with WI-38 (early-passage) cell lysates, the resulting RNP complexes were pulled down using streptavidin-coated beads, and the presence of NF90 in the pulldown RNP complexes was detected by Western blot analysis. As shown in Fig. 5B, NF90 associated with each of the full-length biotinylated 3'UTRs (Fig. 5A), but not with negative control RNAs spanning the nucleolin (*NCL*) mRNA 5'UTR and coding region. These results indicate that NF90 associates *in vitro* with the 3'UTRs of SASP mRNAs.

The influence of NF90 upon the 3'UTRs of SASP mRNAs was assessed using the luciferase reporter constructs (Fig. 5A). The ratio of renilla luciferase (RL, encoded by a reporter transcript bearing each 3'UTR of interest) to firefly luciferase (FL, encoded by an internal control reporter transcript) was set as 1 for the parent vector (psiCHECK2). RL/FL ratios in NF90 siRNA-transfected cells relative to Ctrl siRNA-transfected cells, shown in Fig. 5C (WI-38 cells, *left*; IDH4 cells *right*) indicated substantial increases in reporter expression (except in the psiCHECK-CCL2 and psiCHECK-TNFRSF11B groups), indicating that NF90 repressed the production of the reporter proteins and this repression was alleviated after silencing NF90.

The translation index for each reporter was calculated as the ratio of changes in luciferase activity (RL/FL) relative to the ratio of changes in luciferase mRNA levels (*RL* mRNA*/FL* mRNA). In WI-38 cells, high translation indexes were seen for reporters expressing the 3'UTRs of *CXCL1*, *IL8*,*IL6*, and *CCL16* mRNAs, further suggesting that NF90 repressed their translation. The reporter bearing CSF2 3'UTR also showed an elevated translation index, despite not being a direct target of NF90 (Fig. 2C,D), suggesting that the effect of NF90 on the CSF2 3'UTR was indirect.

### Production of SASP factors is repressed by NF90

**Figure 6:**
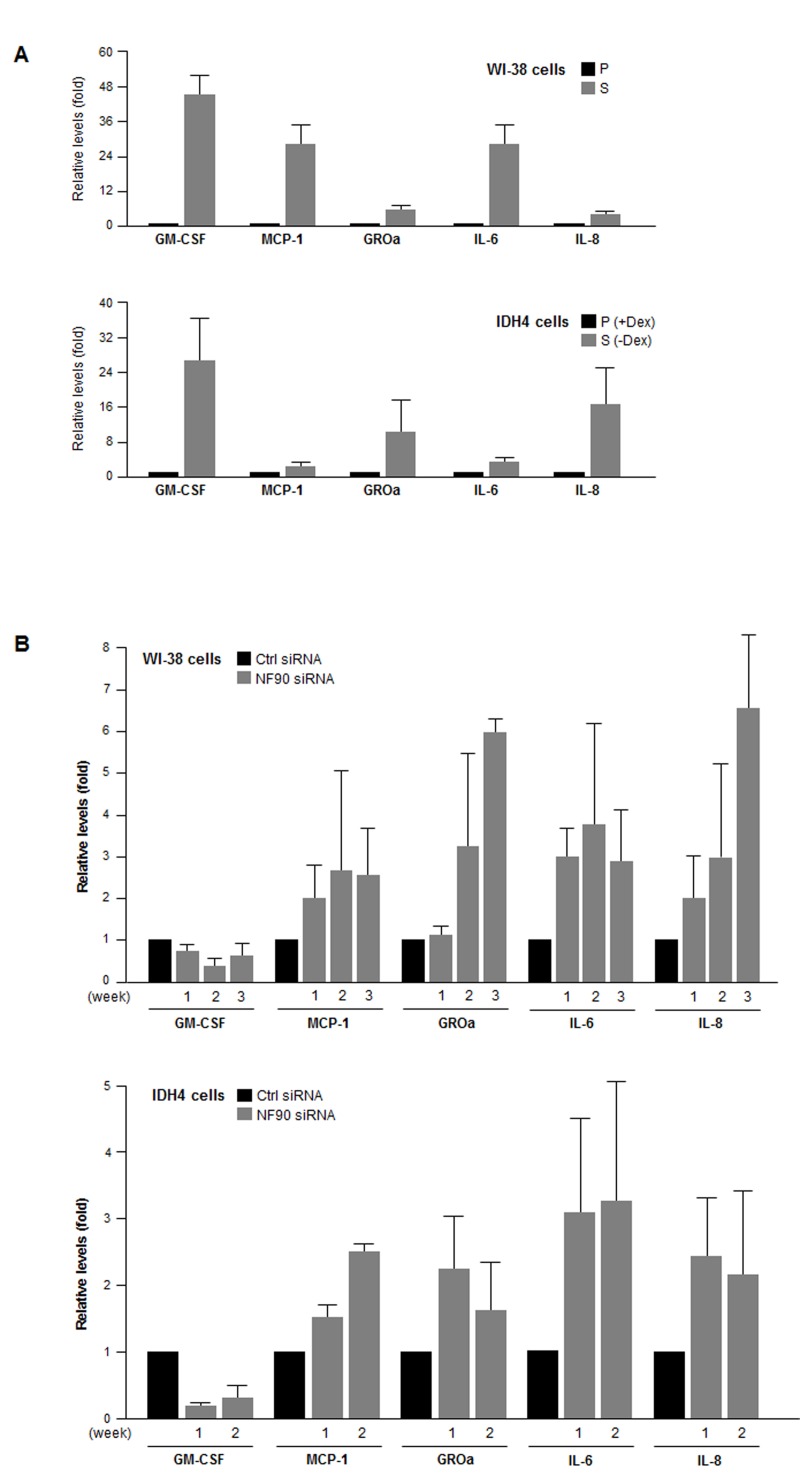
SASP factor abundance as a function of NF90 levels **(A)** The levels of the SASP factors shown (GM-CSF, MCP-1, GROa, IL-6, and IL-8) were measured in supernatants from cultures of WI-38 cells (P, pdl 20; S, pdl 54) and IDH4 cells (+Dex, -Dex for 9 days). **(B)** The levels of the SASP factors were measured in supernatants from cultures of WI-38 cells (pdl 30) and IDH4 cells (+Dex) that were transfected with Ctrl siRNA or NF90 siRNA. SASP factor levels were studied 1–3 weeks after transfection of WI-38 cells (*top*) and 1 and 2 week after transfection of IDH4 cells (*bottom*). In **(A,B)**, data represent the means +SD from 3 independent experiments.

Finally, we examined if the NF90-dependent differen-ces in post-transcriptional regulation of SASP mRNAs were reflected as changes in SASP factor abundance. By multiplex analysis, we first measured the levels of GM-CSF, MCP-1, GRO1, IL-6, and IL-8, encoded by NF90 target transcripts *CSF2* mRNA, *CCL2* mRNA, *CXCL1* mRNA, *IL6* mRNA and *IL8* mRNA, respectively. The levels of these SASP factors were markedly elevated in senescent WI-38 and IDH4 cells relative to early-passage, proliferating cells (Fig. 6A). Importantly, by 1–3 weeks after silencing NF90 through sequential (weekly) siRNA transfections, MCP-1, GROa, IL-6, and IL-8 levels were markedly elevated in proliferating WI-38 and IDH4 populations (Fig. 6B), in line with the finding that NF90 silencing increased the translation of mRNAs bearing the *CCL2*,*CXCL1*, *IL6* and *IL8* 3'UTRs (Fig. 5). By contrast, GM-CSF, encoded by a transcript that was not a target of NF90 (*CSF2* mRNA) was actually lower in the NF90-silenced groups than in Ctrl siRNA cells (Fig. 6B). Taken together, our findings support the notion that NF90 represses the translation of SASP factors GROa, MCP-1, IL-6, and IL-8 through the 3'UTRs of *CCL2*, *CXCL1*, *IL6* and *IL8* mRNAs in proliferating cells, which express high levels of NF90. In senescent cells, NF90 levels decline, which in turn de-represses the biosynthesis of the major SASP factors MCP-1, GROa, IL-6, and IL-8.

## DISCUSSION

We present evidence that the RBP NF90 associates with numerous mRNAs encoding SASP factors. These interactions did not generally influence the steady-state levels of SASP mRNAs, but did appear to repress their translation rates via their respective 3'UTRs. According to this hypothesis, in ‘young’, proliferating human fibroblasts, high levels of NF90 coordinated a state of SASP mRNA repression. By contrast, in senescent fibroblasts, the reduced levels of NF90 led to the de-repression of SASP mRNAs and hence the upregulation of MCP-1, GROa, IL-6, and IL-8 production. The cytokine GM-CSF, encoded by the *CSF2* mRNA, was highly upregulated in senescent cells; surprisingly, however, NF90 did not associate with the *CSF2* mRNA significantly and thus there was no upregulation of GM-CSF levels after silencing NF90 (Fig. 6C,D). The strong reduction in *CSF2* mRNA and GM-CSF protein (Fig. 4,6) after silencing NF90 are likely due to indirect effects of NF90; for example, NF90 may regulate the expression of another RBP that influences *CSF2* mRNA levels, or perhaps NF90, which has DNA-binding activity and can function as a transcription factor [34], might transcriptionally upregulate CSF2 transcription. These possibilities await further study.

Our findings add to a growing body of evidence that SASP is a highly orchestrated trait. Although the SASP comprises enhanced production and secretion of a broad range of factors, such as cytokines, growth factors, and ECM remodeling proteins, a number of ‘master’ regulators of SASP are emerging. Signaling via the MAPK p38 was identified as a global upstream trigger of SASP. At the transcriptional level, activation of NF-κB function, primarily via kinases including p38, led to the coordinated biosynthesis of many SASP mRNAs. Once SASP factors were produced intracellularly, the process of secretion was also coordinated through the levels of IRAK1, a signaling kinase repressed by miR-146a/b. As revealed from our between the processes of transcription and secretion, NF90 functions as a post-transcriptional controller of the fate of SASP mRNAs.

An earlier *en masse* analysis of NF90 target mRNAs in the cervical carcinoma cell line HeLa [33] revealed that NF90 associated with AU-rich elements in the 3'UTRs of target transcripts. These interactions led to the repression of target transcript translation without a significant influence on mRNA stability. As discussed by Kuwano et al. [33], NF90 appeared to reduce translation initiation by blocking the assembly of components of the translation initiation apparatus on a specific mRNA, although NF90 might also recruit one or several types of microRNAs onto the target mRNA, repressing translation via the RISC (RNA-induced silencing complex), as shown for HuR, another RBP [35]. In addition, NF90 is a double stranded (ds)RNA-binding protein (DRBPs); as DRBPs tend to associate with other DRBPs, NF90 might recruit onto target SASP mRNAs other DRBPs implicated in gene silencing, such as DICER, PACT and TRBP, all major components of the RNA silencing machineries [36,37]. Investigating these possibilities also require future work.

Our results have uncovered NF90 as an RBP that jointly regulates subsets of mRNAs encoding proteins that are functionally related (SASP factors). Thus, the regulatory paradigm orchestrated by NF90 might constitute an example of the *post-transcriptional operon* proposed by Keene [38]. According to this model, subsets of mRNAs are regulated coordinately because they share sequence elements that are recognized by a specific RBP. Although the SASP mRNAs identified as NF90 targets in fibroblasts (Fig. 2) bear AU-rich sequences in their 3'UTRs, they were not identified as being NF90 target transcripts in HeLa cells [33], likely because some SASP proteins are not prominently expressed (or even expressed at all) in HeLa cells. These differences underscore the idea that RBPs have different functions in different cells. It is possible that NF90 associates with specific RBPs or ncRNAs in a cell type-dependent manner and these interactions influence the subset of mRNAs that NF90 binds. Other cell type-specific differences may be dictated by post-translational modifications of NF90; although NF90 is phosphorylated by kinases such as PKR, PKC, and AKT [39-42], such modifications have been proposed to affect its RNA-binding function, but they are not fully understood at present. There is extensive crosstalk among p38 and NF90 kinases, but whether they affect NF90 regulation of SASP factor expression also remains to be examined.

One intriguing link between NF90 function and NF-κB activity is provided by MAPK phosphatase MKP-1, whose expression rises through the association of NF90 with the transcript that encodes MKP-1 (*DUSP1* mRNA) [43]. In early-passage cells, high NF90 levels would be predicted to stabilize the *DUSP1* mRNA, increase its translation, and elevate MKP-1 levels; in turn, high MKP-1 levels would be expected to dephosphorylate p38, lowering its activity and hence its stimulatory actions on NF-κB, and further contributing to low SASP gene transcription. In senescent cells, the effect would be reversed: low NF90 maintains low MKP-1 levels, in turn elevating p38 function, which increases NF-κB transcription of SASP genes. Importantly, the mechanisms responsible for elevating NF90 abundance in proliferating fibroblasts and diminishing NF90 levels in senescent fibroblasts are presently unknown.

Finally, silencing NF90 in fibroblasts reduced cell proliferation (Fig. 3B), suggesting that in proliferating fibroblasts, high levels of NF90 are responsible for both repressing SASP factor production and promoting cell division. However, the reduction of NF90 levels in senescent cells did not appear to be sufficient for eliciting senescence, since several senescence markers increased only modestly (p53, p16, p21) and cells did not show elevated ß-galactosidase activity (not shown). Nonetheless, SASP factors like IL-6 and IL-8, induced by depletion of NF90, can operate in an autocrine manner to solidify the growth arrested state of senescent cells [13,14,44]. On the other hand, SASP factors can also stimulate cell transformation, proliferation, and invasion of immortal cells, thereby favoring tumorigenesis [45,46]. By attracting the immune system for elimination of malignant and non-malignant cells, the SASP trait can affect tumor size and wound healing responses [7-9]. Given the importance of the SASP to the biology of senescence and aging, insight into SASP-governing factors such as NF90 provides essential knowledge of age-related physiologic and pathologic changes.

## METHODS

### Cell culture, treatments, transfection, and plasmids

Human WI-38 and IDH4 fibroblasts were cultured in Dulbecco's modified essential medium (DMEM, Invitrogen) supplemented with 10% fetal bovine serum and antibiotics. IDH4 cell culture medium was further supplemented with 1 mM dexamethasone (Dex) for constitutive expression of SV40 large T antigen to suppress senescence and induce proliferation [47]. To induce senescence of IDH4 cells, Dex was removed from the culture media and DMEM was supplemented with charcoal-stripped serum. A control small interfering RNA (Ctrl siRNA) was from Qiagen, and the siRNA directed to NF90 was from Santa Cruz Biotechnologies; the small RNAs were used at 50 nM for WI-38 cells and 10 nM for IDH4 cells. The reporter plasmids psiCHECK2-IL6, -IL8, -CXCL1, -CSF2, -TNFRSF11B, -CCL16, and -CCL2, which were constructed by cloning the corresponding PCR-amplified 3'UTR segments after the *Renilla* (luciferase) RL coding region. Small RNAs and plasmids were transfected with Lipofectamine RNA iMAX and Lipofectamine-2000 (Invitrogen) respectively. Luciferase activity assays were performed using the Dual-Luciferase® Reporter Assay System (Promega).

### Western blot analysis

Whole-cell lysates were prepared using radioimmunoprecipitation assay (RIPA) buffer (10 mM Tris-HCl [pH 7.4], 150 mM NaCl, 1% NP-40, 1 mM EDTA, 0.1% SDS, 1 mM dithiothreitol), separated by electrophoresis in SDS-containing polyacrylamide gels, and transferred onto polyvinylidene difluoride (PVDF) membranes (Millipore). Incubations with primary antibodies recognizing NF90 (mouse monoclonal anti-DRBP76, BD Transduction Laboratories), p21 and AUF1 (Millipore), Nucleolin, HuR (Santa Cruz Biotech) or GAPDH (Abcam) were followed by incubations with the appropriate secondary antibodies conjugated with horseradish peroxidase (HRP) (GE Healthcare) and by detection using enhanced luminescence (GE Healthcare).

### RNA analysis

Total RNA was prepared from whole cells or from RNP immunoprecipitation (IP) samples using TRIzol (Invitrogen). After reverse transcription (RT) using random hexamers and Maxima® Reverse Transcriptase (Thermo), the abundance of transcripts was assessed by real-time, quantitative PCR (qPCR) analysis using the SYBR green PCR master mix (Kapa Biosystems) and gene-specific primer sets (Table 1). RT-qPCR analysis was performed on Applied Biosystems model 7300 and 7900 instruments.

**Table 1:** Sequences of primers used for qPCR analysis. F, forward; R, reverse

*ANG*	F	GGCGTTTTGTTGTTGGTCTT	R	AGTGCTGGGTCAGGAAGTGT
*AXL*	F	TTTCCTGAGTGAAGCGGTCT	R	TCGTTCAGAACCCTGGAAAC
*FGF2*	F	AGCGGCTGTACTGCAAAAAC	R	CTTGATGTGAGGGTCGCTCT
*CCL26*	F	CCTCCTGAGTCTCCACCTTG	R	TGGGAGCAGCTGTTACTGGT
*FAS*	F	ATAAGCCCTGTCCTCCAGGT	R	TGGAAGAAAAATGGGCTTTG
*FGF7*	F	GACATGGATCCTGCCAACTT	R	GGGCTGGAACAGTTCACATT
*CSF2*	F	CTTCCTGTGCAACCCAGATT	R	CAGCAGTCAAAGGGGATGAC
*CXCL2*	F	AACTGCGCTGCCAGTGCT	R	CCCATTCTTGAGTGTGGCTA
*CXCL1*	F	GAAAGCTTGCCTCAATCCTG	R	CACCAGTGAGCTTCCTCCTC
*CCL16*	F	CAGAAAGGCCCTCAACTGTC	R	TGGACAAGTTCCTGGTAGGC
*HGF*	F	CTGGTTCCCCTTCAATAGCA	R	AGCTGCGTCCTTTACCAATG
*ICAM1*	F	GGCTGGAGCTGTTTGAGAAC	R	ACTGTGGGGTTCAACCTCTG
*ICAM3*	F	CTCTGCTGGTCTGCTGTCTG	R	CAAGGCGATTTTCTCAGAGC
*IGFBP1*	F	CTGCCAAACTGCAACAAGAA	R	GAGACCCAGGGATCCTCTTC
*IGFBP2*	F	GAGAAGGTCACTGAGCAGCA	R	ACCTGGTCCAGTTCCTGTTG
*IGFBP4*	F	CCCACGAGGACCTCTACATC	R	ATCCAGAGCTGGGTGACACT
*IGFBP6*	F	CGCGGAAGCTGAGGGCTGTC	R	GTCGTCCTTGGGCGGATGGC
*IL11*	F	ACAGCTGAGGGACAAATTCC	R	AGCTGTAGAGCTCCCAGTGC
*IL13*	F	GTACTGTGCAGCCCTGGAAT	R	CCACCTCGATTTTGGTGTCT
*IL15*	F	GAAGCCAACTGGGTGAATGT	R	ACTTTGCAACTGGGGTGAAC
*IL1B*	F	GGACAAGCTGAGGAAGATGC	R	TCGTTATCCCATGTGTCGAA
*IL6*	F	AAAGAGGCACTGGCAGAAAA	R	TTCACCAGGCAAGTCTCCTC
*IL7*	F	CTCCCCTGATCCTTGTTCTG	R	CCAATTTCTTTCATGCTGTCC
*IL8*	F	CTGCGCCAACACAGAAATTA	R	CTCTGCACCCAGTTTTCCTT
*LEP*	F	GGCTTTGGCCCTATCTTTTC	R	ACCGGTGACTTTCTGTTTGG
*CCL2*	F	TGCTCTGAGAGTTCCCCTGT	R	CTGCCTACACAGGCTGATGA
*CCL8*	F	TCACCTGCTGCTTTAACGTG	R	CACAGCTTCCTTGGGACATT
*CCL13*	F	ATCTCCTTGCAGAGGCTGAA	R	ACTTCTCCTTTGGGTCAGCA
*MIF*	F	AGAACCGCTCCTACAGCAAG	R	GAGTTGTTCCAGCCCACATT
*CCL3*	F	TGCAACCAGTTCTCTGCATC	R	TGGCTGCTCGTCTCAAAGTA
*CCL20*	F	TTTATTGTGGGCTTCACACG	R	GATTTGCGCACACAGACAAC
*OSM*	F	CATCGAGGACTTGGAGAAGC	R	TCAGCCGTGTCTGAGTTGTC
*TNFRSF11B*	F	GGCAACACAGCTCACAAGAA	R	CGCTGTTTTCACAGAGGTCA
*PIGF*	F	TCCACTGGATTGGGAAAGAC	R	ATAACAAGGCCAGCCACGTA
*KITLG*	F	GAAGCAGGGACAGTGGAGAG	R	GCGACTCCGTTTAGCTGTTC
*IL6ST*	F	TGAACGAGGGGAAGAAAATG	R	GTTTTGCTTTGCAATCAGCA
*TNFRSF1A*	F	AAGCGGTCAGTGAGAAGGAA	R	TCTCAGGCCCTTTGAACATC
*TNFRSF1B*	F	ACCAAGTGCCACAAAGGAAC	R	GTTTTCTGAAGCGGTGAAGG
*TIMP2*	F	ACCGTGTGTGACTCCTGTGA	R	GAGTGCAGGCTTGAGTTTCC
*TNFRSF10C*	F	CGCTTCCAACAATGAACCTT	R	GGTCATGGTGCAGGAACTTT
*PLAU*	F	TGTGAGATCACTGGCTTTGG	R	ACACAGCATTTTGGTGGTGA
*18S rRNA*	F	GGAGAGGGAGCCTGAGAAAC	R	TCGGGAGTGGGTAATTTGC

### Immunoprecipitation of ribonucleoprotein (RNP) complexes

Endogenous mRNA-protein RNPs were precipitated from cytoplasmic lysates prepared from early-passage WI-38 or proliferating IDH4 cells. Lysates were prepared in a buffer containing 10 mM Hepes, 100 mM KCl, 5 mM MgCl_2_, 25 mM EDTA, 0.5% IGEPAL, 2 mM DTT, 50 U/ml RNase out and protease inhibitors. Lysates were incubated (4 h, 4°C) with a suspension of protein-A Sepharose beads precoated with anti-NF90 or mouse IgG (BD Transduction Laboratories). The beads were washed with NT2 buffer (50 mM Tris-HCl pH 7.5, 150 mM NaCl, 1 mM MgCl_2_?, 0.05% IGEPAL) and then incubated with NT2 buffer containing RNase-free DNase I (15 min, 30°C), washed with NT2 buffer and further incubated in NT2 buffer containing SDS and Proteinase K to digest proteins bound to the beads. RNA was extracted using phenol and chloroform, precipitated in the presence of glycoblue, and further analyzed by RT-qPCR using the primers listed in Table 1.

### Biotin pulldown analysis

Primers used to prepare biotinylated transcripts spanning SASP mRNAs are listed below. After purification of the template PCR products, biotinylated transcripts were synthesized using MaxiScript T7 kit (Ambion) and whole-cell lysates (200 μg per sample) were incubated with 3 μg of purified biotinylated transcripts for 30 min at room temperature, and then complexes were isolated with Streptavidin-coupled Dynabeads (Invitrogen). Proteins present in the pulldown material were studied by Western blot analysis as described [33].

To synthesize biotinylated 3'UTR RNAs corresponding to the SASP mRNAs, PCR fragments were prepared using forward primers as in that contained the T7 RNA polymerase promoter sequence [(T7), CCAAGCTTCTAATACGACTCACTATAGGGAGA] and the primer sequences in Table 2. The sizes of the resulting RNAs were 427 b for *IL6*, 1250 b for *IL8*, 700 b for *CXCL1*, 314 b for *CSF2*, 816 b for *TNFRSF1*1B, 1067 b for *CCL16*, and 372 b for *CCL2* 3'UTR. Primer pairs to amplify PCR templates for the synthesis of negative RNA controls in biotin pulldown experiments (*NCL* 5'UTR and two CR segments) are indicated in Table 2 and reported previously [48]; the sizes of the resulting RNAs were 142 b for *NCL(5')*, 550 b for *NCL(CR1)*, and 550 b for *NCL(CR2)*.

**Table 2:** Sequences of primers used for qPCR amplification in biotin pulldown experiments. F, forward; R, reverse

*T7-IL6*	F	(T7)CATGGGCACCTCAGATTG	R	GCTGAATTTTTTAAAATGCCATTT
*T7-IL8*	F	(T7)AAAAATTCATTCTCTGTGG	R	ACTTTGACAACAAATTATATT
*T7-CXCL1*	F	(T7)CCAGAAGGGAGGAGGA	R	CTATAAGTGCTTTATTTTTATATTC
*T7-CSF2*	F	(T7)GACCGGCCAGATGAGG	R	TAGAAGCATATTTTTAATAATAATT
*T7-TNFRSF11B*	F	(T7) CTGGAAATGGCCATTGAG	R	AAAAAATAATTTGAAAACTTTAATA
*T7-CCL16*	F	(T7)TGACCAGGCTTTAGTGGA	R	TGTCAAATAAGGGAGTTTTATTTAT
*T7-CCL2*	F	(T7) ACACTCACTCCACAACCC	R	GTACAAAAATATATTTATTTGGTGT
*T7-NCL(5')*	F	(T7)CTTTCGCCTCAGTCTCGAGCTCT	R	TGATGGCGGCGGAGTGTGAAGCGG
*T7-NCL(CR1)*	F	(T7)ATGGTGAAGCTCGCGAAG	R	AGGCAGGGGCAGCAGC
*T7-NCL(CR2)*	F	(T7)GAACCAGCAGCGATGAAA	R	AATCCACATAACCAAATTTCCTA

### Measurement of SASP factors

The concentration of SASP factors (GM-CSF, MCP-1, GROa, IL-6, and IL-8) in the culture supernatant was analyzed by using multiplex assay, following the manufacturer's instructions (Biorad Laboratories, Hercules, CA). Data were normalized and expressed as pg per ml per cell per 24 h.

## Abbreviations

CR: coding region
pdl: population doubling
NF90: nuclear factor 90
RBP: RNA-binding protein
RNP: ribonucleoprotein
UTR: untranslated region
